# Supplementary material for: Content-rich biological network constructed by mining PubMed abstracts
Source: BMC Bioinformatics. 2004 Oct 8;5:147. doi: 10.1186/1471-2105-5-147 (PMC528731; doi:10.1186/1471-2105-5-147)
Supplement: Additional File 5 — The original Chilibot query results of the term "long-term potentiation (LTP)" and 22 other terms, limiting the latest references analyzed to the years 1990, 1995, 2000, and 2004. [file 1471-2105-5-147-S5.bz2 › chilibotAdditionalFile5/ltp2000/html/CREB_CAMKIV.html]

 


 **CREB** and **CAMKIV** 
  
Found 15 abstracts in PubMed,  **15 abstracts were retrieved and analyzed**.  


---

 Search Google  |
 PDF files only 
|  EDU domain only 

---

**Interactive relationship** (e.g. stimulation, inhibition, etc)

- In T lymphocytes,  **CaMKIV**  will phosphorylate  **CREB**  in response to signals that result in T cell activation.  Ref: 9238860 Recent Prog Horm Res, 1997
- It was also found in these studies that Ser133 of  **CREB**  is essential for its activation by  **CaMKIV** .  Ref: 7958915 Genes Dev, 1994
- Transcriptional activation of the BZLF1 promoter by  **CaMKIV**  Gr is dependent on the  **CREB**  AP1 binding element ZII and is greatly augmented by the calcium calmodulin dependent phosphatase calcineurin.  Ref: 9261377 J Virol, 1997
- The  **CaMKIV**  mediated gene induction requires the activity of  **CREB**  ATF family members but is independent of PKA activity.  Ref: 8065343 Mol Cell Biol, 1994
- The results indicate that  **CaMKIV**  is much more potent than CaMKII in activating  **CREB**  in three different cell lines.  Ref: 7958915 Genes Dev, 1994
- When Ser142 was mutated to alanine,  **CREB**  was activated by CaMKII, as well as by  **CaMKIV** .  Ref: 7958915 Genes Dev, 1994
- Extracellular signal regulated kinase,  **CaMKIV** , and p38 MAPK were also the kinases involved in  **CREB**  Ser 133 phosphorylation induced by CD3 CD28.  Ref: 11123304 J Immunol, 2001
- Stimulation of T lymphocytes results in a rapid increase in intracellular calcium concentration calcium i that parallels the activation of calcium calmodulin dependent protein kinase IV  **CaMKIV** , a nuclear enzyme that can phosphorylate and activate the cyclic adenosine monophosphate cAMP response element binding protein  **CREB** .  Ref: 9596578 Science, 1998
- Mutagenesis studies and phosphopeptide mapping analysis demonstrated that in vitro,  **CaMKIV**  phosphorylates  **CREB**  at Ser133 only.  Ref: 7958915 Genes Dev, 1994
- Our results suggest that  **CaMKIV**  may represent a physiologically relevant  **CREB**  kinase in T cells.  Ref: 9171236 Mol Endocrinol, 1997
- Because both CaMKII and  **CaMKIV**  can phosphorylate  **CREB** , we pursued further the mechanism by which CaMKII and  **CaMKIV**  differentially regulate  **CREB**  activity.  Ref: 7958915 Genes Dev, 1994
- **CaMKIV**  Gr deficient mice exhibited impaired neuronal cAMP responsive element binding protein  **CREB**  phosphorylation and calcium  **CREB**  dependent gene expression.  Ref: 10964952 J Neurosci, 2000
- Nuclear  **CaMKIV**  regulates transcription through phosphorylation of several transcription factors, including  **CREB** .  Ref: 10366852 Trends Biochem Sci, 1999
- We further showed that  **CaMKIV**  could be involved in the calcium dependent expression of the immediate early gene c fos, probably via phosphorylation of the transcription factor  **CREB** .  Ref: 8898857 Biochim Biophys Acta, 1996

**Parallel relationship** (e.g. studied together, co-existance, homology, etc.)

- However, despite impaired LTP and  **CREB**  activation,  **CaMKIV**  Gr deficient mice exhibited no obvious deficits in spatial learning and memory.  Ref: 10964952 J Neurosci, 2000
- **CaMKIV**  dependent activation of  **CREB**  is a key event in the expression of genes involved in the processes of T cell activation and neuronal long term potentiation.  Ref: 10191497 Biometals, 1998
- In studying the signaling pathways by which synaptic inputs control the phosphorylation state of cyclic AMP responsive element binding protein  **CREB**  and determine expression of CRE regulated genes, we found two important calcium calmodulin CaM regulated mechanisms in hippocampal neurons a CaM kinase cascade involving nuclear  **CaMKIV**  and a calcineurin dependent regulation of nuclear protein phosphatase 1 activity.  Ref: 8980227 Cell, 1996
- A late phase of cerebellar long term depression requires activation of  **CaMKIV**  and  **CREB** .  Ref: 10433267 Neuron, 1999
- These results indicate that the activation of  **CaMKIV**  and  **CREB**  are necessary to establish a late phase of cerebellar LTD.  Ref: 10433267 Neuron, 1999
- **CaMKIV**  is a potent mediator of calcium induced gene expression, primarily through its ability to phosphorylate and activate transcription factors such as  **CREB** .  Ref: 10191497 Biometals, 1998
- In Jurkat T cells, inhibition of PP2A activity by small t antigen enhanced activation of  **CREB**  mediated transcription by  **CaMKIV** .  Ref: 9596578 Science, 1998
- Exposure of neurons to BDNF stimulates  **CREB**  phosphorylation and activation via at least two signaling pathways by a calcium calmodulin dependent kinase IV  **CaMKIV**  regulated pathway that is activated by the release of intracellular calcium and by a Ras dependent pathway.  Ref: 9390517 Neuron, 1997
